# The Effectiveness of Platelet-Rich Plasma in the Management of Rotator Cuff Tears: A Systematic Review and Meta-Analysis

**DOI:** 10.7759/cureus.98132

**Published:** 2025-11-30

**Authors:** Suvank Rout, Souvagya Rout

**Affiliations:** 1 Trauma and Orthopaedics, Topiwala National Medical College and Bai Yamunabai Laxman Nair Charitable Hospital, Mumbai, IND; 2 Trauma and Orthopaedics, Colchester General Hospital, Colchester, GBR; 3 Trauma and Orthopaedics, First Faculty of Medicine, Charles University, Prague, CZE

**Keywords:** prp injection, rotator cuff arthropathy, rotator cuff tears, shoulder, shoulder mobility sports injury

## Abstract

Platelet-rich plasma (PRP) injections have been proposed to enhance tendon healing and alleviate symptoms in rotator cuff tears, but clinical evidence of their efficacy remains mixed. This systematic review synthesizes the evidence from randomized controlled trials (RCTs) on PRP’s effectiveness for rotator cuff tears. We conducted a systematic review following the Preferred Reporting Items for Systematic Reviews and Meta-Analyses guidelines. We searched PubMed, Excerpta Medica Database (EMBASE), and the Cochrane Library from inception through March 2025 for RCTs comparing PRP injection with placebo, corticosteroid injection, or no adjunct therapy in adults with partial- or full-thickness rotator cuff tears. Primary outcomes included pain [Visual Analog Scale (VAS)], functional scores [American Shoulder and Elbow Surgeons (ASES)], Constant-Murley score, and tendon healing on imaging. Thirty-six studies (approximately 2000 patients) met the inclusion criteria. PRP generally produced significant short-term pain relief, with pooled data showing lower VAS scores at six weeks, three months, six months, and one year compared with controls (all P<0.05). In terms of function, PRP often improved ASES and Constant-Murley scores during the first three to six months. However, long-term functional gains were inconsistent, and some trials reported no between-group differences. Regarding tendon healing, a large meta-analysis of arthroscopic repair RCTs (n=1359) found a significantly lower retear rate with PRP (16.5% vs 23.6%, P=0.002), while other trials found no difference. Current evidence suggests that PRP injection provides significant short-term pain reduction and functional improvement in adults with rotator cuff tears and may enhance tendon healing when used as a surgical adjunct. However, considerable heterogeneity in PRP protocols and mixed long-term results mean its definitive superiority over standard care is not firmly established. PRP may be considered a potential adjunct, but further high-quality, standardized RCTs are needed.

## Introduction and background

Rotator cuff tears are a common cause of shoulder pain and dysfunction. The prevalence of rotator cuff tears increases with age, affecting approximately 9.7% of individuals aged 20 or younger and up to 30% of those over 60 years of age [1]. Tears may or may not present with symptoms. Although acute trauma can cause tears, most result from chronic, degenerative tendinopathy, which is an overuse disorder characterized by progressive microtrauma, cellular changes, and matrix degradation [2]. Conservative management, including physical therapy and non-steroidal anti-inflammatory drugs (NSAIDs), and surgery are mainstays of treatment, yet outcomes can remain suboptimal because of persistent pain, functional limitations, and failed tendon healing [3]. 

Surgical repair remains an option for suitable patients but carries limitations, including postoperative pain and a significant risk of retear [2]. For large tears, retear or repair failure rates can reach 8-94% [2]. The fundamental biological challenge lies in the tendon tissue’s poor healing capacity, particularly at the tendon-to-bone insertion site, or “enthesis” [2]. Limited vascularity in this region restricts the influx of reparative cells and growth factors necessary for robust healing [4]. The inability to regenerate the original tissue architecture and the body’s slow, incomplete repair response often result in chronic pain, weakness, and limited functionality [4]. To address this poor healing capacity, biological therapies such as platelet-rich plasma (PRP) have emerged as leading candidates for biological augmentation in soft tissue healing [5]. PRP is an autologous blood product concentrate composed of alpha granules (platelet granules) that release key growth factors [6]. Platelets release cytokines such as platelet-derived growth factor, transforming growth factor-beta, and vascular endothelial growth factor, which promote angiogenesis, cell proliferation, and tissue regeneration [7]. Ultrasound-guided PRP injection into the subacromial space or tendon may improve shoulder function, reduce pain, and lower retear rates in rotator cuff pathology [8]. However, findings remain inconsistent, and no consensus has been established [9].

Therefore, the critical questions are not simply whether PRP works but under what specific conditions, for which patient populations, and using which formulations, as well as for what clinical goals PRP may provide measurable benefit. We conducted a systematic review to evaluate PRP injections versus placebo, corticosteroid injection, or no adjunct therapy in adults with partial- or full-thickness rotator cuff tears, focusing on functional outcomes, tendon healing, and pain. 

## Review

Method

Search Strategy

We conducted a systematic literature search using PubMed, Excerpta Medica Database (EMBASE), and the Cochrane Central Register of Controlled Trials from inception to March 2025, employing the terms “platelet-rich plasma,” “PRP,” “rotator cuff,” and “randomized controlled trial.” We also performed backward chaining by screening reference lists from identified articles and prior reviews. 

Inclusion Criteria

We included randomized controlled trials (RCTs) comparing PRP injections with control interventions such as placebo (e.g., saline injection), no injection, standard physical therapy, or other active treatments (e.g., corticosteroid injection) in adults over 18 years diagnosed with partial- or full-thickness rotator cuff tears confirmed by imaging (MRI or ultrasound). We also included studies using PRP as an adjunct to arthroscopic repair. Outcomes included pain [visual analog scale (VAS)] and shoulder-specific functional scores [e.g., Constant-Murley score, American Shoulder and Elbow Surgeons (ASES)] [10,11,12].

Exclusion Criteria

**Figure 1:**
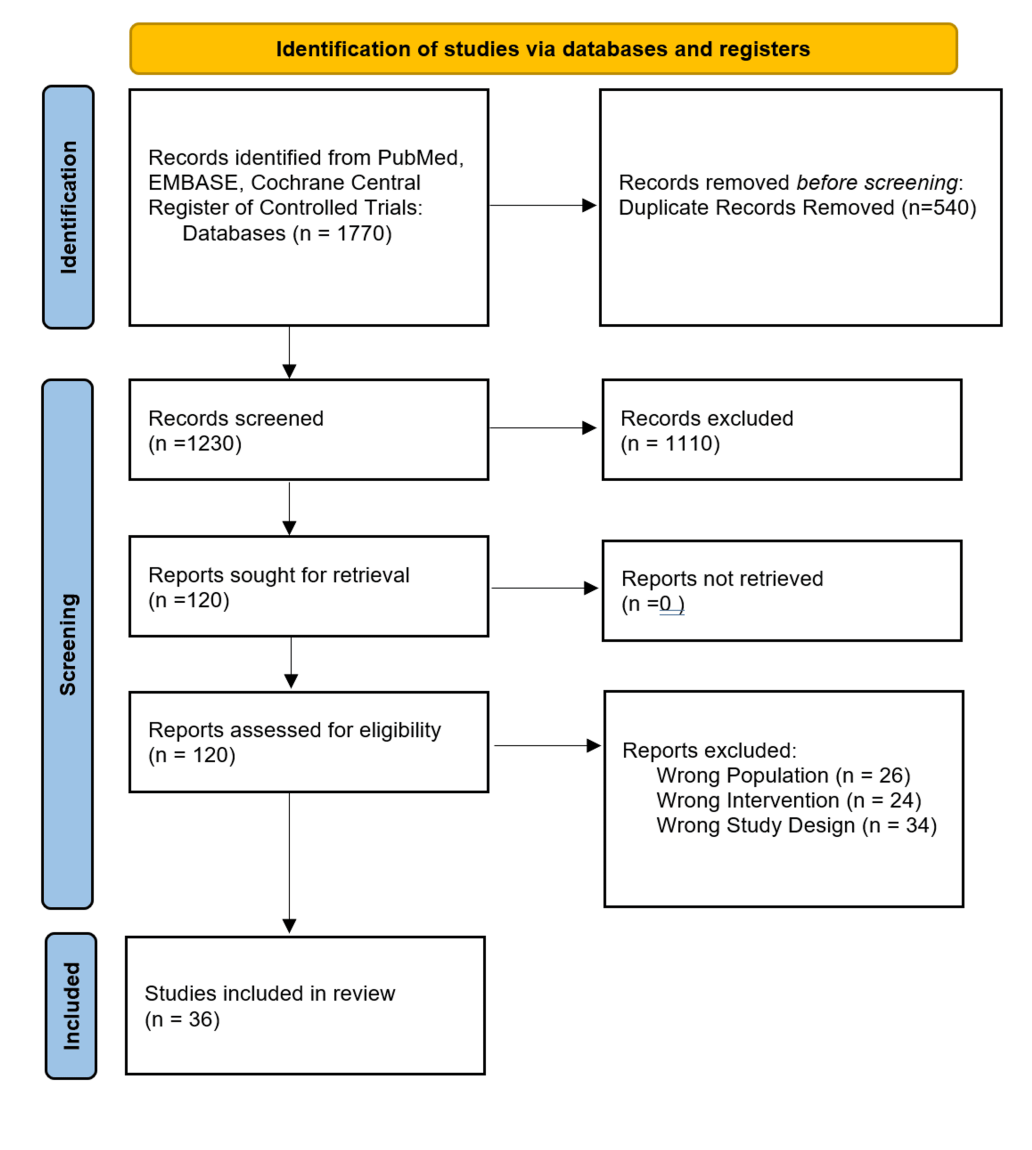
PRISMA Flow Diagram of the Study Selection Process PRISMA: Preferred Reporting Items for Systematic Reviews and Meta-Analyses

We excluded studies without a comparative group, studies focused on other shoulder pathologies, non-English publications, and those with less than three months of follow-up. The literature search identified 36 studies involving approximately 2000 patients. We followed Preferred Reporting Items for Systematic Reviews and Meta-Analyses (PRISMA) flow diagram guidelines, as Figure 1 shows.

Data Synthesis 

Following the search, two authors independently screened all retrieved titles and assessed the potentially eligible articles against the eligibility criteria. Any disagreements at either stage were resolved through discussion and by consulting a third author. The literature revealed substantial clinical heterogeneity in patient populations, PRP protocols, and control interventions. Therefore, a quantitative meta-analysis was not feasible for all outcomes. Where possible, we summarized pooled estimates from existing meta-analyses; otherwise, we provided a qualitative synthesis of the included RCTs. The literature search identified 36 Studies involving approximately 2000 patients.

Quality Assessment

**Figure 2:**
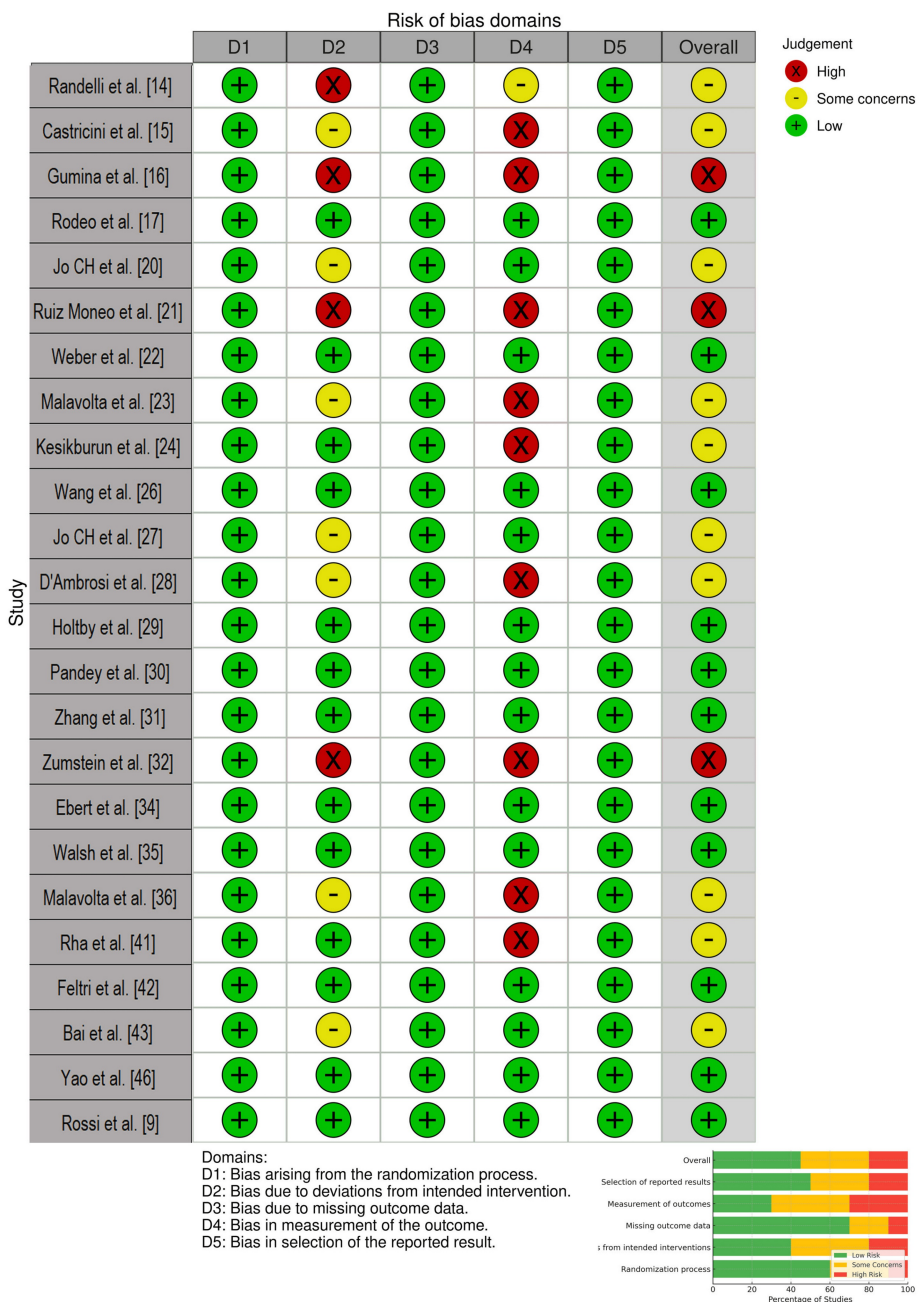
Risk of Bias Assessment of all the Randomised Control Trials with the Cochrane RoB2 Tool

Risk of bias assessment for the randomised control trials in the studies was evaluated using the Cochrane Risk of Bias 2 (RoB 2) tool. Two authors independently performed this assessment against the five domains: randomization process, deviations from intended interventions, missing outcome data, measurement of outcomes, and selection of reported results (Figure 2). 

Results

The initial literature search yielded 1,230 studies. After title and abstract screening, 1,110 were excluded. One hundred and twenty full-text articles underwent eligibility assessment, with 84 being excluded. Twelve RCTs examined the nonsurgical management of partial-thickness rotator cuff tears, assessing the efficacy of PRP compared with hyaluronate, steroids, or saline. The remaining studies investigated PRP use during arthroscopic rotator cuff repair. Seventeen trials used leukocyte-rich (LR-PRP), twelve trials used leukocyte-poor (LP-PRP), and four trials did not specify leukocyte concentration. Table 1 summarizes study characteristics. Geographically, the studies were distributed across Asia (15 studies), Europe (10 studies), the Americas (nine studies), and the Middle East (two studies). In non-operative protocols, most studies delivered PRP via ultrasound-guided subacromial injection, whereas surgical trials typically applied PRP gel intraoperatively at the tendon-bone interface.

**Table 1:** Summary of Randomized Controlled Trials Evaluating Platelet-Rich Plasma (PRP) in Rotator Cuff Tears (n=36) PRP: platelet rich plasma, MRI: magnetic resonance imaging, VAS: visual analogue score, ASES: American Elbow and Shoulder Score, UCLASS: University of California at Los Angeles Shoulder Score, SST: simple shoulder test score.

SURGICAL – PRP GEL APPLIED INTRAOPERATIVELY	CLINICAL QUESTION	DELIVERY / SETTING	NUMBER OF PATIENTS IN THE STUDY	PRIMARY OUTCOMES ASSESSED	KEY CONCLUSION
Cai et al. (Meta-analysis) [13]	Does PRP improve structural and clinical outcomes after cuff repair?	Pooled intraoperative studies	837	MRI Retear rate, UCLASS, Constant Score	PRP reduced retear rate and improved early function.
Randelli et al. [14]	Does intraoperative PRP improve tendon healing and function after rotator cuff repair?	PRP applied at the tendon–bone interface during arthroscopy	53	MRI Healing, Constant Score, VAS, UCLASS	PRP reduced early postoperative pain but showed no long-term benefit in healing or function.
Castricini et al. [15]	Does PRP augmentation enhance repair integrity and outcomes?	PRP gel applied intraoperatively	88	Constant Score, MRI retear rate	No improvement in tendon healing or function.
Gumina et al. [16]	Does a leukocyte-rich platelet membrane enhance tendon healing?	Membrane applied intraoperatively	76	ASES, Constant Score, SST Score, Ultrasound	No structural or clinical advantage with leukocyte-rich PRP.
Rodeo et al. [17]	Does PRFM improve tendon-bone healing?	PRFM applied during arthroscopic repair	79	MRI healing, ASES score	No improvement in healing; possible increase in stiffness.
Chahal et al. (Meta-analysis) [18]	Does PRP improve clinical or structural outcomes across studies?	Pooled intraoperative RCTs	1,105	MRI Retear rate, Constant Score, SST, ASES, UCLASS	No consistent benefit; high study heterogeneity.
Zhang et al. (Meta-analysis) [19]	Are platelet-rich products necessary for arthroscopic repair?	Pooled intraoperative RCTs	1,105	MRI Retear rate, Constant Score, SST, ASES, UCLASS	PRP showed no clear efficacy; evidence inconclusive.
Jo et al. [20]	Can PRP improve the healing of large or massive cuff tears?	PRP gel applied intraoperatively	48	VAS, ASES, Constant Score, UCLA, DASH, SST, MRI	PRP reduced retear rate and improved early pain relief.
Ruiz-Moneo et al. [21]	Does PRGF improve tendon integrity and healing?	Injected intraoperatively at the repair site	63	Retear rate, Constant Score, UCLASS	No significant improvement in healing or function.
Weber et al. [22]	Does PRFM enhance tendon healing after repair?	PRFM at the tendon–bone junction	60	MRI healing, ASES score, UCLASS, ASES, SST	No significant differences in healing or outcomes.
Malavolta et al. [23]	Does PRP improve tendon integrity and reduce pain after repair?	PRP gel applied intraoperatively	54	MRI healing, Constant Score, VAS, UCLASS	No difference in retear rate or functional results.
Kesikburun et al. [24]	Does PRP injection provide superior pain relief compared to saline in chronic partial tears?	Non-operative – ultrasound-guided PRP injection	200	VAS, Constant Score	PRP did not significantly outperform placebo.
Warth et al. (Meta-analysis) [25]	Does PRP enhance repair integrity and shoulder function?	Pooled intraoperative studies	1,427	MRI retear rate, Constant Score, SST, ASES, UCLA	PRP reduced retear rate but did not improve function.
Wang et al. [26]	Do postoperative PRP injections accelerate tendon healing and recovery?	PRP injected after surgery	60	MRI healing, VAS, ROM	No benefit of postoperative injections on healing or function.
Jo et al. [27]	Does PRP improve outcomes in medium-to-large tears?	PRP gel applied during arthroscopy	74	MRI Retear rate, VAS, Constant Score, ASES, UCLASS, SST,	PRP reduced retears and improved early function.
D’Ambrosi et al. [28]	Does intraoperative PRP improve tendon healing?	PRP gel applied intraoperatively	40	Ultrasound, VAS, Constant Score	Trend toward fewer retears, but not statistically significant.
Holtby et al. [29]	Is PRP beneficial in small-to-medium tears?	PRP gel applied during repair	82	Constant Score, MRI Retear Rate, VAS, ASES, Constant Score	No significant improvement in outcomes.
Pandey et al. [30]	Does PRP with moderate platelet concentration enhance healing?	Gel applied intraoperatively	102	MRI integrity, VAS, Constant Score, ASES, UCLA	PRP reduced retears and improved functional outcomes.
Zhang et al. [31]	Does PRP improve outcomes in double-row repairs?	Gel applied intraoperatively	60	MRI healing, Constant Score, VAS	PRP reduced retears and improved early function.
Zumstein et al. [32]	Does leukocyte-rich fibrin influence long-term tendon healing?	L-PRF matrix applied intraoperatively	35	MRI healing, Constant Score, SST	No difference in long-term structural healing.
Fu et al. (Meta-analysis) [33]	What is the pooled effect of PRP or fibrin matrix on cuff repair?	Multiple intraoperative RCTs	1,232	Retear, clinical outcomes	PRP reduced retear rate but did not consistently improve function.
Ebert et al. [34]	Do delayed postoperative PRP injections improve healing?	PRP injected post-surgery	53	MRI, ASES, Constant Score	No additional benefit.
Walsh et al. [35]	Does fibrin-based PRP reduce retears and improve healing?	PRFM applied intraoperatively	72	MRI, ASES, VAS, SST	PRFM reduced retears and improved early recovery.
Malavolta et al. [36]	Are the benefits of PRP sustained at long-term follow-up?	PRP applied intraoperatively	51	MRI, Constant Score, VAS, UCLASS	No long-term advantage observed.
Han et al. (Meta-analysis) [37]	Is PRP an effective adjunct for cuff repair?	Pooled intraoperative RCTs	1,844	MRI retear rate, Constant Score, SST, ASES, UCLA	PRP reduced retear rates and early pain.
Hurley et al. (Meta-analysis) [8]	What is the efficacy of PRP and PRF in arthroscopic cuff repair?	Pooled intraoperative RCTs	1,593	ASES, MRI	PRP/PRF reduced retears; greatest benefit in double-row repairs.
Zhao et al. (Meta-analysis) [38]	Does leukocyte-poor PRP improve repair outcomes?	LP-PRP applied intraoperatively	1,139	Retear rate, VAS, ASES, UCLASS, Constant Score	LP-PRP significantly reduced retears and pain.
Ryan et al. (Meta-analysis) [39]	Does PRP supplementation improve healing and clinical outcomes?	Pooled intraoperative RCTs	2,046	Retear rate, Constant Score, SST, ASES, UCLA,	PRP reduced retears and improved short-term function.
Villarreal-Villarreal et al. (Meta-analysis) [40]	Does PRP enhance outcomes in double-row cuff repairs?	PRP applied intraoperatively	1,326	Retear rate, Constant Score, ASES, SST, VAS	PRP significantly reduced retears in double-row repairs.
Rha et al. [41]	Is PRP more effective than dry needling for partial-thickness tears?	Non-operative – ultrasound-guided PRP injection	40	VAS, Constant Score	PRP provided superior pain relief and functional recovery at 6 months.
Feltri et al. [42]	Does PRP produce a clinical benefit in patients with rotator cuff disorders	Surgical and Non Surgical - Pooled RCT data	2423	MRI retear rate, Constant Score, ASES, SST, VAS	PRP does not improve clinical results in patients with rotator cuff disorders, but reduces the retear rate
Bai et al. [43]	Does PRP concentration influence healing outcomes?	Injection during surgery	80	Retear, Constant Score	Higher-concentration LP-PRP improved healing quality.
Shen et al. (meta-analysis of 21 RCTs) [44]	What is the pooled effect of PRP augmentation in arthroscopic repair?	Surgical – pooled RCT data	1359	VAS, ASES, Constant Score, retear	PRP reduced retear risk (~30%) and modestly improved pain/function.
Desouza et al. (meta-analysis of 12 RCTs) [45]	What is the pooled effect of PRP injections for partial tears?	Non-operative – pooled RCT data	762	VAS, ASES, Constant Score	PRP improved short-term pain and function but not long-term outcomes.
Yao et al. [46]	Does intraoperative injection of leukocyte-rich or leukocyte-poor PRP versus control improve tendon healing and functional outcomes?	Surgical – intraoperative PRP	150	VAS, ASES, UCLASS, Constant Score, MRI retear	No significant difference in 12-month functional or structural outcomes among groups; LR-PRP showed superior ASES at 3 and 6 months.
Rossi et al. [9]	Does PRP injection improve pain and function for rotator cuff tendinopathy vs control (corticosteroid)?	Non-operative – Double Blind RCT with PRP injection vs corticosteroid	50	VAS, ASES, Constant Score, MRI retear	Subacromial PRP injection in patients with rotator cuff tendinopathy showed significantly superior and sustained pain-relieving and functional improvements compared with one corticosteroid subacromial injection, assessed by 4 patient-reported outcome scales at the 12-month follow-up

Impact on Pain Outcomes

**Figure 3:**
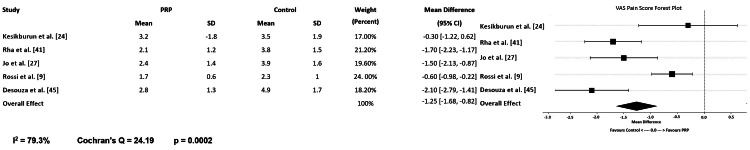
Forrest Graph for Pain scores (VAS) at 6 Months - Non-operative Studies [9,24,27,41,45], Forrest Plot created using Microsoft Excel (Microsoft Corporation, Redmond, United States of America). (I^2=heterogeneity amount).

Across included trials and meta-analyses, PRP generally produced greater pain reduction than comparators. Desouza and Shetty found PRP to be effective for both short- and long-term pain reduction in partial-thickness tears [45]. In Desouza and Shetty’s analysis of 12 RCTs, standardized mean differences in VAS at six weeks and six months were -2.04 and -2.26, respectively, favoring PRP (both P≤0.01). Rossi et al. found mean 12-month VAS scores of 1.68 (SD 0.6) with PRP compared with 2.3 (SD 1.0) with corticosteroid (P<0.001) [45]. Hurley et al. noted lower VAS scores in the PRP group at 30 days postoperatively (2.9 vs. 4.3) and at final follow-up (1.2 vs. 1.4) [8,12]. Shen et al., in their pooled analysis of randomized controlled trials, found that for partial-thickness tears, PRP significantly improved VAS scores at six weeks, three months, six months, and 12 months post-injection [44]. Castricini et al. and Pandey et al. observed that PRP-treated patients showed reduced early postoperative discomfort and inflammation [15,30]. Jo et al. found in their study that PRP produced greater pain reduction and functional gain at six months when compared to corticosteroid injections for non non-operative group [27]. However, some studies did not confirm these benefits. Kesikburun et al., in their large randomized controlled trial, compared PRP to saline for subjects with chronic rotator cuff tears. They found no significant difference in VAS scores between PRP and saline groups at one-year follow-up [24]. Additionally, in the meta-analysis of nonoperative studies, PRP significantly improved short term pain outcomes with a pooled mean difference of -1.25 [95% xinfidence interval (CI): -1.68 to -0.82] as noted on the visual analogue scale at six months (Figure 3) In summary, most evidence indicates that PRP reduces pain more effectively than a placebo or a steroid, especially within the first six to 12 months. PRP may accelerate early pain relief, but does not provide a lasting advantage beyond two years. 

Impact on Functional, Clinical, and Recovery Outcomes

**Figure 4:**
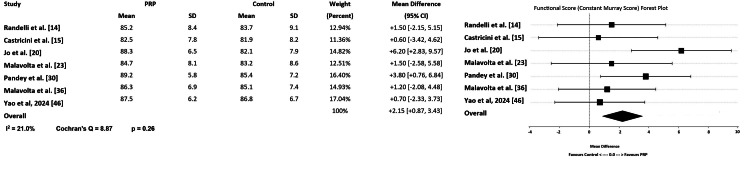
Forrest Graph representing the Functional Outcomes (Constant-Murley Score) at 12 Months [14,15,20,23,30,36,46], Forrest Plot created using Microsoft Excel (Microsoft Corporation, Redmond, United States of America). (I^2=heterogeneity amount).

Functional improvement from PRP remains less conclusive. Several meta-analyses reported significant short-term improvements, but long-term results were inconsistent. Randelli et al. and Hurley et al. found that PRP improved short-term shoulder function assessed by Constant-Murley score and University of California at Los Angeles (UCLA) activity scores in subjects who had partial tears and were treated nonoperatively [8,10,11,12,14]. Rha et al. noted that PRP significantly increased ASES and Constant-Murley scores at three to six months [41]. Desouza and Shetty reported standardized mean differences of +1.21 (ASES) and +2.01 (Constant-Murley score), favoring PRP short-term (P<0.05) [45]. The above functional impact and statistical improvement raise some doubts. Jo et al. noted that although there was statistical improvement in outcomes, none of the changes reached the minimally clinically important difference (MCID) [27]. MCID represents the smallest change in an outcome score that a patient perceives as beneficial and that prompts a clinician to consider a change in management. It serves as a bridge between statistical significance and real-world clinical relevance. Conversely, Desouza et al. reported that despite pain relief, long-term functional recovery was not significant, and Cai et al. also found no significant long-term functional improvement in their analysis. Both studies focused on patients treated nonoperatively [9,44]. When analyzing studies involving patients who underwent arthroscopic rotator cuff repair, Shen et al. noted in their meta-analysis that while PRP improved short-term UCLA and Constant-Murley scores, it did not provide an advantage in the ASES score, even at 12 months of follow-up [44]. Jo et al. corroborated this finding, concluding that PRP improved short- but not long-term functional outcomes after arthroscopic repair of full-thickness tears [27]. A modest improvement of +2.15 (95% CI: +0.87 to +3.43) was noted for the Constant-Murley scores, which did not consistently exceed the minimal clinically important difference at the 12-month follow-up (Figure 4).

Effects of PRP on Tendon Healing (on Imaging) and Retear Rates

**Figure 5:**
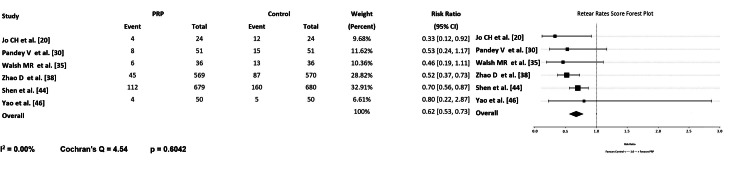
Forrest Graph Showing Retear Rates (Surgical Studies) [20,30,35,38,44,46], Forrest Plot created using Microsoft Excel (Microsoft Corporation, Redmond, United States of America). (I^2=heterogeneity amount).

In their meta-analysis of 21 surgical randomized control trials, Hurley et al. found a significantly lower retear rate with intra-operative PRP injection against the control group [8]. Several other meta-analyses strongly supported this finding. Pandey et al. demonstrated that intraoperative PRP application at the tendon-bone interface significantly improved tendon healing integrity on follow-up MRI without increasing complication rates [30]. Zhao et al. and D'Ambriosi et al. similarly observed lower retear rates in both primary and revision arthroscopic repairs augmented with PRP [28,38]. Hurley et al. [8] confirmed these results in large-scale RCTs, noting that PRP significantly reduced the risk of repair failure compared with control repair. A three-arm RCT by Yao et al. compared LR-PRP, LP-PRP, and a control and found an overall retear rate of approximately 8% at 12 months, with no significant difference between the groups [46]. Furthermore, Pandey et al. confirmed that PRP significantly decreases long-term retear rates but emphasized that the functional benefit of this structural improvement is often absent [30]. Cai et al. documented that in nonsurgical settings, PRP significantly reduced the tear size for small-to-medium partial tears at the one-year MRI follow-up [13]. Histologic and imaging evidence indicate that PRP promotes organized collagen deposition and reduces early gap formation, thereby improving the mechanical stability of the repair [18-21]. The meta-analysis of surgical studies consistently demonstrated that PRP reduces retear rates with a pooled risk ratio (RR) of 0.62 [95% CI: 0.53-0.73], suggesting a roughly 38% lower risk of retear compared to the control group (Figure 5). The consistent reduction in retear rates across multiple independent cohorts strengthens the argument for PRP as a biologic enhancer of tendon healing rather than merely a symptomatic therapy. Although retear rates are lower, not all studies demonstrate proportional improvements in function, implying that structural integrity alone may not fully determine long-term outcomes. Variability in PRP formulation, concentration, and delivery methods likely contributes to the heterogeneity of results across trials.

Risk of Bias Assessment 

The authors noted that the selected studies generally exhibited a moderate level of methodological quality, with notable heterogeneity amongst the studies. We noted that the overall distribution of bias indicated that 45% of studies had a low risk of bias, 35% moderate risk of bias, and 20% were deemed to have a high risk of bias. Performance and detection bias were the most frequent sources of high risk, particularly in surgical trials where blinding of PRP application was not feasible. Despite these limitations, most trials maintained low attrition rates and adequate randomization procedures.

Discussion

Discrepancies in the evidence likely arise from significant clinical heterogeneity. Factors such as PRP preparation (leukocyte content, platelet concentration), injection technique (subacromial vs. intratendinous), tear characteristics (size, chronicity), and comparator type (corticosteroid vs. saline) vary widely between studies, complicating direct comparisons [42,43].

PRP does not represent a single standardized product. These variations can result in different biological properties and lead to varied clinical outcomes. One of the most debated variables is the concentration of leukocytes in the final PRP product. Leukocytes can have both proinflammatory and anti-inflammatory effects, and their precise role in healing remains unclear. Studies report conflicting results regarding LR-PRP and LP-PRP formulations. Jo et al., Pandey et al., and Shen et al. found that LP-PRP produced significantly better functional outcomes (Constant-Murley scores) than LR-PRP [27,30,44]. Conversely, Wang et al. and Warth et al. reported no significant functional benefit despite improved healing rates [25,26]. Similarly, the timing and mode of delivery influence patient outcomes. Kesikburun et al. reported that the long-term functional benefit of PRP injections in tendinopathy was not significant, but the improvement in pain was substantial for patients who received PRP injections [24]. This supports the concept that nonoperative PRP primarily provides pain control. In nonoperative PRP trials, this review found that symptom relief was greater with PRP than with corticosteroids or placebo, as Rossi et al. and Cai et al. noted [9,13]. In the context of surgical augmentation for full-thickness tears, PRP functions as a short-term biological bridge. This early protection, accompanied by faster pain relief, provides a temporary advantage in the short term. Over the long term, however, the structural and functional outcomes of PRP-augmented and standard repairs are not significantly different. The use of platelet-rich fibrin is not recommended because current evidence shows that it provides no benefit and increases operative time [8].

Limitations and future recommendations

This review has limitations, primarily reflecting those of the available evidence. The included studies exhibited substantial heterogeneity in PRP formulation, dosing, and control interventions. Many trials were small and carried a risk of bias, particularly in blinding. Publication bias is also possible because small positive studies are more likely to be published. Most studies have short- to mid-term follow-up durations (≤24 months), with limited data on long-term durability or outcomes beyond two years. The variability in imaging outcome reporting prevented a comprehensive pooled analysis for tendon healing.

The most significant barrier to progress in orthobiologics research is the lack of standardization. Adopting a consistent classification and reporting system for PRP composition, rather than using the generic term “PRP,” is essential. To resolve current inconsistencies in the literature, researchers must conduct well-designed RCTs using standardized formulations and long-term follow-up-ideally over a period of 5 years-to evaluate durability and clinical relevance. 

## Conclusions

After a comprehensive review of the literature, it is clear that PRP is not a panacea for rotator cuff pathology. Current evidence demonstrates that subacromial PRP injection can significantly reduce pain and improve shoulder function in the short term, particularly compared with corticosteroids. However, routine use in all patients cannot yet be recommended, as the magnitude of functional improvement remains modest and inconsistent, and the structural benefit of reduced retear rates does not consistently translate into meaningful, patient-reported functional improvements. The present synthesis suggests that PRP can be a valuable adjunct in the management of rotator cuff pathology when appropriately selected and applied. In non-operative cases, PRP offers a safe alternative to corticosteroids, providing longer-lasting pain relief and promoting intrinsic tendon repair without deleterious tissue effects. In surgical repair, PRP may enhance tendon-bone integration and reduce early structural failure which is supported by the lower retear rates 12 months postoperatively.

Given the contradictory evidence, future research must focus on standardizing PRP preparation methods, extending follow-up durations, and conducting well-designed RCTs. PRP appears to function as a short-term adjunct, but its long-term effectiveness remains inconclusive, preventing its adoption as standard practice.
